# A Unique Case of Autoimmune Retinopathy Associated with Anti-Alpha-Enolase Antibodies

**DOI:** 10.1155/2011/371324

**Published:** 2011-12-19

**Authors:** Paul A. Kurz, Rachel E. Reem, Daryl E. Kurz, Richard G. Weleber

**Affiliations:** ^1^Dayton VA Medical Center, Dayton, OH 45428, USA; ^2^Havener Eye Institute, The Ohio State University, 915 Olentangy River Road, Columbus, OH 43212, USA; ^3^Roudebush VA Medical Center, Indianapolis, IN 46202, USA; ^4^Department of Ophthalmology, Indiana University, 702 Rotary Circle, Indianapolis, IN 46202-5133, USA; ^5^Casey Eye Institute, Oregon Health & Science University, 3375 SW Terwilliger Boulevard, Portland, OR 97239-4197, USA

## Abstract

*Background*. We report a case of autoimmune retinopathy associated with anti-alpha-enolase antibodies with unique manifestations. *Methods*. A case report. *Results*. A 30-year-old male experienced recurrent, primarily peripheral visual field disturbances and minimal photopsia, with interval symptom resolution. Fundus changes subsequently developed in areas corresponding to the previous visual field symptoms. Electroretinogram showed bilaterally symmetric abnormalities of light-adapted responses and suggested loss of photoreceptor function. Only anti-alpha-enolase antibodies were detected on Western blot. Our patient noted cutaneous symptoms at the time of both episodes of visual symptoms, but not in the interim. Biomicroscopy revealed subtle small reddish spots in areas of the peripheral retina corresponding to the areas of the patient's visual field where he noted symptoms. To our knowledge these reddish spots have not been reported in autoimmune retinopathy and may clinically support *in vitro* and *in vivo* evidence that anti-alpha-enolase antibodies may target photoreceptors. *Conclusions*. Our patient demonstrates some unique features adding to the known characteristics of autoimmune retinopathy associated with anti-alpha-enolase antibodies. As more cases are reported, further understanding of the features and pathophysiology of this rare condition will hopefully be elucidated.

## 1. Introduction

Autoimmune retinopathy (AIR) (nonparaneoplastic) and cancer-associated retinopathy (CAR) (paraneoplastic) are immunologically mediated retinal degenerations associated with antibodies directed against retinal proteins. The literature varies in diagnostic criteria for AIR and firm establishment of this diagnosis is challenging [1–3]. It has been proposed that the presence of antirecoverin antibodies (Ab) or anti-alpha-enolase Ab (AAEAb) is diagnostic of AIR in the context of symptoms of acute or subacute vision loss, abnormal electroretinogram (ERG), and exam findings of diffuse retinal atrophy with little or no pigment deposition [2, 3]. Photopsia, lack of cell or flare, multiple bands of antiretinal antibody activity on Western blot, electronegative ERG, personal or family history of autoimmune disease, and rapid progression are reportedly common in these patients as well [2, 3]. Progression of AIR associated with AAEAb is typically slower than progression of AIR associated with anti-recoverin Ab [1].

Approximately 30% of all patients with anti-retinal Ab have AAEAb [4]. AAEAb are less predictive of associated neoplasm than anti-recoverin Ab yet have been found in patients who have skin cancer or cancers with endocrine features [4]. CAR in patients with AAEAb typically develops months to years after discovery of the malignancy [1].

While it has been suggested that AIR responds to corticosteroids or immunosuppression, the value of treatment remains uncertain. Treatment is least effective for nonparaneoplastic AIR [3]. One analysis found that corticosteroid and immunosuppressive therapy were clinically ineffective in cases of AIR associated with AAEAb [1]. We report a case of AIR associated with AAEAb that has not required treatment and exhibits unique manifestations.

## 2. Case Presentation

A 30-year-old Chinese male presented with episodic visual disturbance. Symptoms began 18 months earlier with black spots in the inferior visual field (VF) OS. The spots were more noticeable in dim lighting and moved centrally over 2 weeks. He denied flashes, pain with eye movement, redness, photophobia, nausea, vomiting, or headache. Throughout the initial episode, visual acuity (VA) remained 20/20 in each eye. Exam was remarkable only for mild retinal vessel attenuation and perivascular pigment deposition in the inferior periphery OS. Color vision, fluorescein angiogram, MRI brain/orbits, RPR, and FTA-Abs were normal. ERG revealed bilaterally symmetric abnormalities of light-adapted responses, including reduced 30-hertz flicker amplitudes with normal implicit times, and normal dark-adapted, rod-mediated responses. The maximum scotopic rod-cone response exhibited decreased a-wave amplitude suggesting loss of photoreceptor function.

The black spots gradually resolved over 4 months without treatment. He remained asymptomatic, until returning one year later noting sudden onset of dark spots in his inferotemporal VF OS and superior VF OD. He denied flashes, pain, photophobia, nausea, vomiting, and headache. Fluorescein angiogram and Goldmann VF were within normal limits. He was referred to our clinic 2 weeks later.

**Figure 1:**
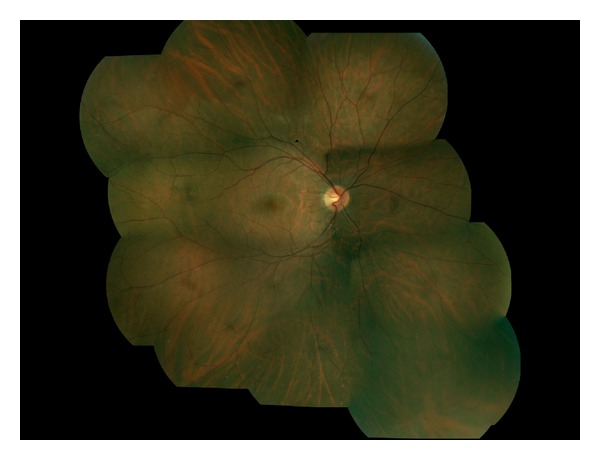
Color fundus photo montage OD. Exam was remarkable for mild vessel narrowing with areas of hypopigmentation most notable in the superotemporal periphery, although also noted in the inferior/inferonasal periphery. Peripapillary depigmentation (more evident temporally and less extensive than the ring of depigmentation noted OS) is present. Biomicroscopy revealed subtle reddish spots, not consistent with hemorrhage, within areas corresponding to the areas of the visual field where he described symptoms. These subtle red spots were most notable in the far inferonasal periphery and are difficult to appreciate in the photos.

**Figure 2:**
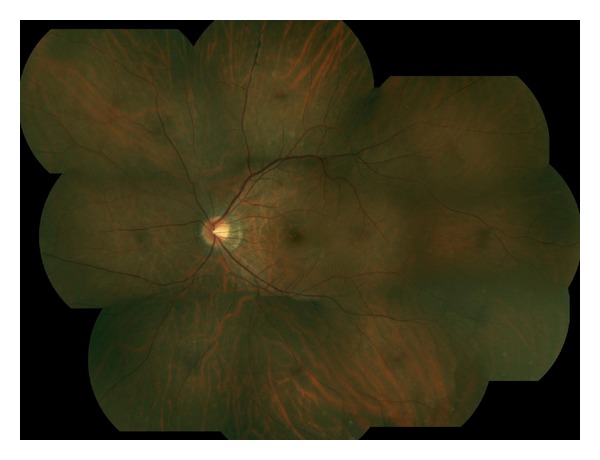
Color fundus photo montage OS. Vessel narrowing with perivenular pigment deposition and surrounding hypopigmentation is noted in the superior periphery. On exam, perivascular pigment deposition was also present in the far inferior periphery (too peripheral to be captured in the photos). Hypopigmentation is also noted in the inferior periphery. A ring of peripapillary depigmentation is evident. Subtle reddish spots (difficult to appreciate in the photos) were noted on biomicroscopy in the far inferior periphery and far superonasal periphery.

By then, his black spots had become pinkish. He reported 5 hours of intermittent flashes 2 days prior, noticeable only against a white background. VA remained 20/20 in each eye. Fundus findings are shown. (Figures 1 and 2). All symptoms had resolved without treatment 2 months following the second episode's onset.

Dermatologic findings that he described as itchy “hives” or “welts” occurred with both episodes of visual symptoms. During his first episode, they recurred on his upper and lower extremities then turned purple and resolved over 3 weeks. They did not recur once the visual symptoms resolved. One week prior to the onset of his second episode, they developed all over his body, with associated fever, myalgias, abdominal ache, and fatigue. He denied nausea, vomiting, diarrhea, or bloody stools. Symptoms resolved in 24 hours. Allergy testing was negative. He denied insect bites. Home inspection for bedbugs was negative. A dermatologist never examined him at these times.

A hypersensitivity to mosquito bites in Asians has been reported in chronic active Epstein-Barr Virus (EBV) infection [5]. Some suggest that AZOOR may be related to Epstein-Barr Virus (EBV) [6]. We tested for EBV Ab. IgM was negative. IgG was positive. These results, typical for much of the population, suggest past infection with EBV. He had no clinical signs of chronic active EBV infection.

A diagnosis of Acute Zonal Occult Outer Retinopathy (AZOOR) was considered; however, a workup for AIR was performed. Western blot testing for antibodies to approximately 1200 retinal proteins revealed only AAEAb. Age-appropriate malignancy workup, including chest X-ray, complete blood count with differential and platelets, comprehensive metabolic panel, and physical by his primary care physician were within normal limits. Dermatology consult revealed no malignancies. There was no personal or family history of autoimmune disease, including asthma, thyroid disease, or inflammatory bowel disease. Testing for antinuclear Ab, p and c antinuclear cytoplasmic Ab, myeloperoxidase Ab, proteinase 3 Ab, rheumatoid factor, and antiphospholipid Ab was negative. Erythrocyte sedimentation rate and C-reactive protein were within normal limits.

## 3. Discussion

AIR has been suggested to represent a secondary complication of other conditions, including AZOOR, with the diagnosis of AIR established with detection of AAEAb in the context of these symptoms [2]. Our patient shares features of AIR and AZOOR, yet differs from the typical presentations of both.

Retinal pigment changes corresponding to location of VF symptoms, mild retinal vessel narrowing, sparing of central vision, and reduced amplitude of 30-hertz flicker responses are consistent with AZOOR [7, 8]. Our patient developed superior perivascular pigment deposition OS between the first and second episodes, presumably as a result of the process causing the inferior VF symptoms during the first episode. Our patient's differences from the typical AZOOR presentation included scotomas more noticeable in dim illumination, lack of prominent photopsias, symmetry of the ERG between the 2 eyes, absence of delay in the 30-hertz flicker responses, and complete symptom resolution [7, 8]. Our patient's symptoms were initially noted peripherally. VA, Amsler grid, color vision, and Goldmann VF remained normal. Onset was sudden. Typically, AIR associated with AAEAb begins with evidence of central visual dysfunction and onset is subacute or chronic [1]. Although gradual progression is a hallmark of AIR, our patient has not demonstrated progressive vision loss [2]. Stepwise vision loss may occur in AZOOR [7].

One reported patient, who presented with scintillations, good VA, and was initially diagnosed with AZOOR, was later found to have AAEAb. Unlike our patient, that patient had a normal fundus exam, asymmetric electroretinogram and VF findings, and persistent VF defects [1].

Evidence suggests that AAEAb are intrinsically pathogenic. AAEAb from patients with CAR have been shown to induce apoptosis of retinal cells *in vitro* and *in vivo*, by targeting antigens in the ganglion cell and inner nuclear layers, and also photoreceptors [1]. Biomicroscopy of our patient disclosed subtle reddish spots within peripheral retinal areas corresponding to the areas of his visual field where he described visual disturbances. In acute macular neuroretinopathy (one of the AZOOR-complex diseases), reddish lesions appear at the outer sensory retina, due to outer retinal damage. Spectral domain optical coherence tomography demonstrated photoreceptor outer segment abnormalities that can explain blind spot enlargement in AZOOR-complex diseases [9]. Perhaps the AAEAb in our patient are damaging outer retinal cells, creating similar reddish lesions, with subsequent photoreceptor loss.

In the context of his symptoms and clinical findings, our patient can be diagnosed with AIR associated with AAEAb. Anti-retinal antibody levels fluctuate over time and can serve as a biomarker for disease activity associated with worsening of visual symptoms [10]. Perhaps the episodic nature of our patient's symptoms resulted from fluctuating AAEAb levels. Perhaps the skin reactions that our patient had were triggers for or signs of increased immune system activity, including increased levels of AAEAb and associated symptomatic episodes. This single case demonstrates unique findings and raises some interesting questions. As more cases of AIR are accumulated and studied, hopefully a better understanding of this rare condition will be achieved.

##  Conflict of Interests

The authors report no conflicts of interest. The authors alone are responsible for the content and writing of the paper.
